# Endothelial Responses to ICS/LABA Combinations Are Determined Primarily by the Corticosteroid Component

**DOI:** 10.3390/ijms27157045

**Published:** 2026-08-06

**Authors:** Bassam Redwan, Christian Biancosino, Amir Mehdizadeh-Shrifi, Felix Nikolaus Lennartz, Stefan Fischer, Karsten Wiebe, Marion Schael, Patrick Zardo, Sabina Janciauskiene, Heiko Golpon

**Affiliations:** 1 Department of Thoracic Surgery, Knappschaft Kliniken Gelsenkirchen Buer, 45894 Gelsenkirchen, Germany; 2 Helios University Hospital Wuppertal, University of Witten/Herdecke, 42283 Wuppertal, Germany; christian.biancosino@helios-gesundheit.de; 3 Department of Respiratory Medicine, Hannover Medical School (MHH), 30625 Hannover, Germany; amirmehdizadehshrifi@yahoo.com (A.M.-S.); lennartz.felix@mh-hannover.de (F.N.L.); schael.marion@mh-hannover.de (M.S.); janciauskiene.sabina@mh-hannover.de (S.J.); golpon.heiko@mh-hannover.de (H.G.); 4 Department of Thoracic Surgery and Lung Assist, Klinikum Ibbenbüren, 49477 Ibbenbüren, Germany; s.fischer@mathias-stiftung.de; 5 Department of Thoracic Surgery, University Hospital of Münster, 48149 Münster, Germany; karsten.wiebe@ukmuenster.de; 6 German Center for Lung Research (DZL), Biomedical Research in End-Stage and Obstructive Lung Disease (BREATH), 30625 Hannover, Germany; zardo.patrick@mh-hannover.de; 7 Department of Cardiothoracic, Transplantation and Vascular Surgery (Division of Thoracic Surgery), Hannover Medical School (MHH), 30625 Hannover, Germany

**Keywords:** budesonide, formoterol, fluticasone propionate, salmeterol, endothelial network formation, pulmonary microvascular endothelial cells, endothelial apoptosis, VEGF, IL-8, MCP-1, emphysema, COPD, inhaled corticosteroids, endothelial repair, efferocytosis

## Abstract

The pulmonary microvasculature plays an essential role in maintaining alveolar integrity, but the direct effects of inhaled corticosteroids (ICS) on pulmonary endothelial cell function remain poorly understood. We investigated the effects of budesonide, formoterol, and their combination on endothelial network formation, apoptosis, migration, proliferation, and efferocytosis using human pulmonary microvascular endothelial cells (HPMEC-ST1.6R), A549 alveolar epithelial cells, and phagocytic cell models. Budesonide, alone or combined with formoterol, altered endothelial network formation on Matrigel by reducing total tubule length and the number of junctions while increasing tubule thickness, indicating reduced branching complexity. Budesonide also decreased epithelial secretion of the pro-angiogenic mediators VEGF, IL-8, and MCP-1. An independent set of experiments showed that fluticasone propionate similarly reduced VEGF, IL-8, MCP-1, TNF-α, and IL-6, supporting a class effect of corticosteroids on the epithelial secretome. Despite reducing angiogenic responses, budesonide protected endothelial cells from apoptosis, reduced caspase-3/7 activity, accelerated wound closure without affecting proliferation, preserved endothelial spheroid integrity, and enhanced efferocytosis. Formoterol alone had minimal effects and did not consistently enhance budesonide responses. Together, these in vitro findings suggest that, in the cell models studied, pulmonary endothelial responses to the ICS/LABA combinations tested are determined predominantly by the corticosteroid component, which suppresses pro-angiogenic mediator secretion while preserving endothelial survival and repair. As an exploratory cell-based study, these observations are hypothesis-generating and require confirmation in primary cells and in vivo models before clinical inferences can be drawn.

## 1. Introduction

Chronic obstructive pulmonary disease (COPD) is defined clinically by chronic bronchitis, chronic bronchiolitis and emphysema, and remains a leading cause of morbidity and mortality worldwide. While protease–antiprotease imbalance and chronic inflammation have long dominated conceptual models of emphysema, accumulating evidence over the past two decades has established programmed death of alveolar structural cells as an additional and potentially upstream mechanism of alveolar destruction [1,2]. In particular, the pulmonary capillary endothelium appears central to disease initiation and progression: experimental blockade of vascular endothelial growth factor (VEGF) signaling induces rapid endothelial apoptosis and airspace enlargement in rodents, while human emphysematous lungs exhibit increased endothelial and epithelial apoptosis accompanied by reduced VEGF and VEGFR2 expression [3,4].

VEGF is highly expressed in the normal lung and functions as a critical endothelial survival factor via phosphatidylinositol 3′-kinase/Akt-dependent signaling [5,6,7]. Disruption of VEGF signaling, either by ligand withdrawal or receptor inhibition, triggers endothelial apoptosis that is amplified by oxidative stress and ceramide accumulation, linking impaired endothelial maintenance to progressive alveolar destruction [8,9]. Given the structural dependence of alveolar integrity on an intact capillary network, endothelial injury is increasingly considered as a driver rather than a consequence of emphysema pathogenesis [3]. A second key determinant of COPD pathobiology is defective efferocytosis. Efficient clearance of apoptotic cells by alveolar macrophages and epithelial cells terminates inflammation and promotes resolution through mediators such as TGF-β1 [10,11,12]. In contrast, impaired efferocytosis leads to secondary necrosis, persistence of pro-inflammatory stimuli, and ongoing tissue injury. Defective efferocytosis has been consistently demonstrated in COPD and is thought to contribute to chronic inflammation and failed resolution [10,11,12].

Inhaled corticosteroid (ICS)/long-acting β2-agonist (LABA) combinations, such as budesonide/formoterol, reduce exacerbation frequency and improve clinical outcomes in COPD, with evidence suggesting complementary anti-inflammatory actions of the two drug classes [13,14,15,16]. ICS have also been shown to suppress VEGF expression and pathological angiogenesis in airway disease, particularly in asthma-related airway remodeling [17,18,19]. However, whether these agents directly modulate pulmonary microvascular endothelial survival, angiogenic behavior, and repair programs remains unclear, as does the apparent paradox between anti-angiogenic effects and preservation of endothelial viability in emphysema.

Here, we used complementary in vitro cell-based models including human pulmonary microvascular endothelial cells (HPMEC-ST1.6R) [20], A549 alveolar epithelial cells, and professional and non-professional phagocytes to examine the cellular effects of budesonide, formoterol, and their combination on (i) endothelial network formation (Matrigel tube formation and three-dimensional spheroids), (ii) endothelial viability and caspase-dependent apoptosis, (iii) proliferation and migration, (iv) epithelial–macrophage secretion of angiogenesis-associated mediators, and (v) efferocytosis. To determine whether suppression of the epithelial secretome is a general effect of corticosteroids rather than a property specific to budesonide, we analyzed an independent dataset using fluticasone propionate and salmeterol in epithelial and macrophage models. Similar reductions in inflammatory and pro-angiogenic mediators supported a corticosteroid class effect. Overall, our findings demonstrate a coordinated endothelial response characterized by altered network organization, preservation of endothelial viability under stress, and enhanced repair, while providing no consistent evidence that the LABA contributed additional biological activity. Together, our results suggest that, under these in vitro conditions, the endothelial effects of the ICS/LABA combinations tested are driven primarily by the corticosteroid component.

## 2. Results

### 2.1. Phagocyte Pre-Incubation with Budesonide Enhances Phagocytic Clearance of Apoptotic Cells

**Figure 1:**
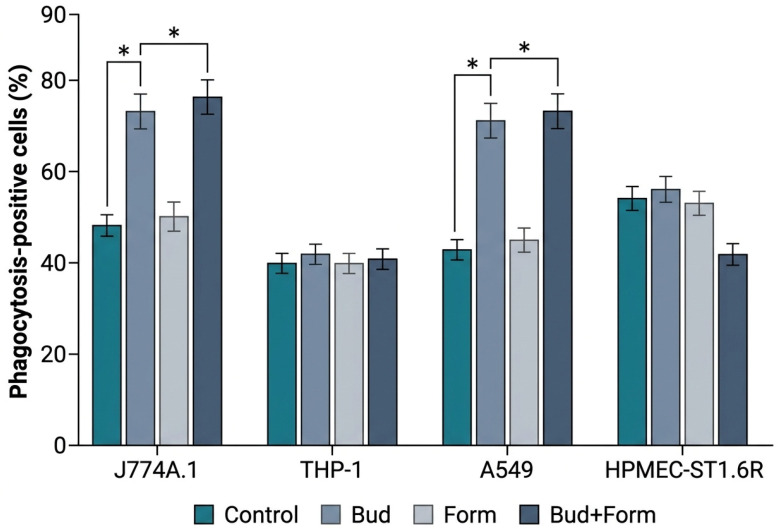
Pre-incubation with budesonide enhances efferocytosis in macrophages and alveolar epithelial cells. Phagocytes (J774A.1, THP-1-derived macrophages, A549, and HPMEC-ST1.6R cells) were pre-incubated for 24 h with budesonide (Bud), formoterol (Form), or their combination (Bud + Form; 10^−6^ M each) before exposure to apoptotic Jurkat T cells. Bars represent the percentage of phagocytosis-positive cells (mean ± SEM, *n* = 4). Budesonide alone and in combination with formoterol significantly increased efferocytosis by J774A.1 macrophages and A549 cells, whereas the combination reduced uptake by HPMEC-ST1.6R cells. Formoterol alone had no significant effect. * *p* < 0.05 versus control (one-way ANOVA followed by Student–Newman–Keuls post hoc test).

Pre-incubation of phagocytes for 24 h with budesonide and/or formoterol (10^−6^ M) before exposure to apoptotic Jurkat T cells revealed that budesonide, either alone or in combination with formoterol, significantly increased the proportion of phagocytosis-positive J774A.1 macrophages (≈48% in control to ≈73–76%) and A549 alveolar epithelial cells (≈43% to ≈71–73%; *p* < 0.05, *n* = 4). THP-1-derived macrophages showed no significant change. In contrast, the budesonide/formoterol combination reduced apoptotic cell uptake by HPMEC-ST1.6R endothelial cells (from ≈54% to ≈42%), Formoterol alone had no effect, and the combination provided no additional activity beyond budesonide (Figure 1).

### 2.2. Budesonide Reduces Secretion of Angiogenesis-Associated Mediators by Alveolar Epithelial Cells

**Figure 2:**
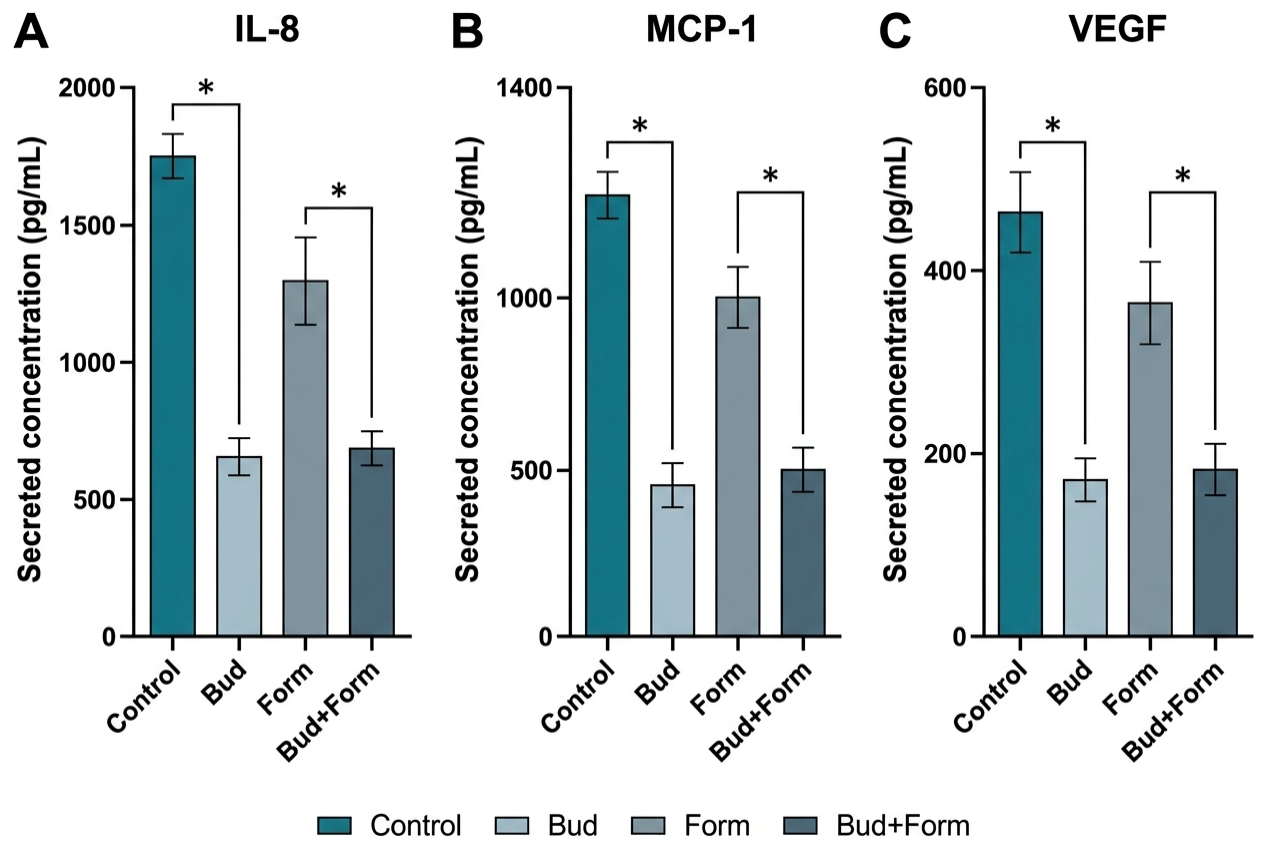
Budesonide suppresses the secretion of IL-8 (**A**), MCP-1 (**B**), and VEGF (**C**) by alveolar epithelial cells. A549 cells were treated for 24 h with budesonide (Bud), formoterol (Form), or their combination (Bud + Form; 10^−6^ M each). Concentrations of IL-8, MCP-1, and VEGF in culture supernatants were quantified by ELISA. Data are presented as mean ± SEM (*n* = 3). Budesonide significantly reduced the secretion of all three mediators, whereas formoterol exerted a weaker effect. The combination produced responses comparable to budesonide alone, indicating no additional activity of the combination. * *p* < 0.05 versus control.

Pre-incubation of A549 alveolar epithelial cells with budesonide, and to a lesser extent formoterol, significantly reduced the secretion of IL-8 (≈1800 to ≈650 pg/mL), MCP-1 (≈1400 to ≈480 pg/mL), and VEGF (≈460 to ≈170 pg/mL), as determined by enzyme-linked immunosorbent assay (ELISA) (* *p* < 0.05, *n* = 3) (Figure 2). These coordinated changes affect mediators that contribute to both inflammatory recruitment and vascular remodelling, defining a modified epithelial secretory phenotype. The budesonide/formoterol combination produced effects comparable to budesonide alone (Figure 2), indicating that formoterol did not provide any additional inhibitory effect.

### 2.3. Budesonide Protects Pulmonary Microvascular Endothelial Cells from Apoptosis

**Figure 3:**
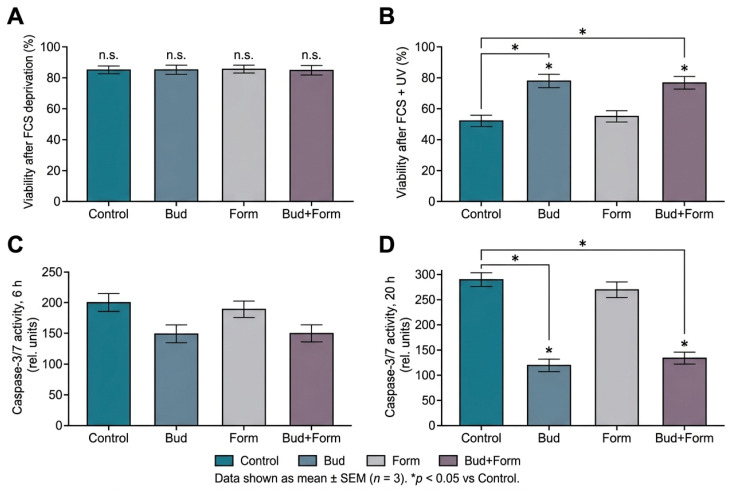
Budesonide preserves endothelial viability and reduces caspase-3/7 activity. (**A**) HPMEC-ST1.6R cell viability after 6 h serum deprivation alone (no significant difference between groups). (**B**) Cell viability after 6 h of serum deprivation combined with UV irradiation; budesonide (Bud) and budesonide/formoterol (Bud + Form) significantly increased the proportion of viable cells compared with UV-exposed control. (**C**) Caspase-3/7 activity at 6 h and (**D**) at 20 h, both reduced by Bud and Bud + Form compared with UV-treated control. Treatments were applied at 10^−6^ M. Data are shown as mean ± SEM (*n* = 3). * *p* < 0.05 versus UV-treated control.

To model endothelial apoptosis, HPMEC-ST1.6R cells in suspension were exposed to fetal calf serum (FCS)-free medium, with or without UV irradiation, and viability was assessed by Hoechst-33342/Sytox-Green fluorescence microscopy. Serum deprivation alone for 6 h caused only a modest reduction in viability (≈85% viable as compared to controls), with no significant drug effect (Figure 2A). In contrast, UV irradiation markedly reduced viability (control ≈52%), and this loss was significantly prevented by budesonide (≈78%) and budesonide/formoterol combination (≈77%; *p* < 0.05, *n* = 3) (Figure 3B). Consistent with reduced apoptosis, caspase-3/7 activity slightly decreased by budesonide alone and budesonide/formoterol at 6 h (Figure 3C) and significantly decreased at 20 h (control ≈290 to ≈115–135 relative units, respectively) (Figure 3D). Formoterol had no effect and, in combination with budesonide, did not enhance the effects of budesonide alone (Figure 3).

### 2.4. Budesonide Accelerates Endothelial Wound Closure Without Affecting Proliferation

**Figure 4:**
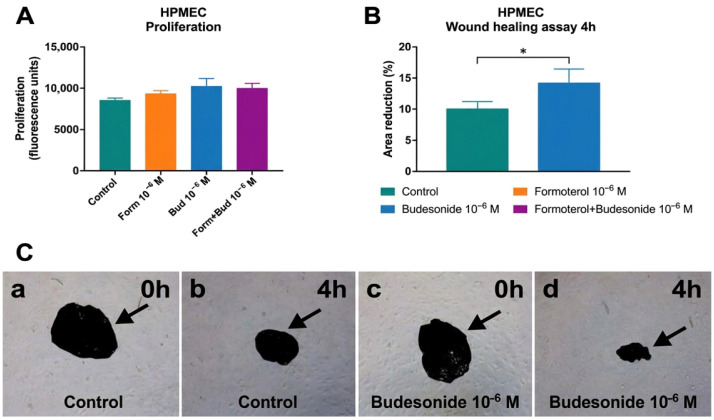
Budesonide accelerates endothelial monolayer repair. (**A**) Proliferation (CyQUANT) showing a non-significant increase. (**B**) Wound-healing assay: reduction in wound area at 4 h, significantly greater with budesonide. (**C**) Representative phase-contrast images at 0 h and 4 h for control and budesonide. Mean ± SEM, *n* = 3. * *p* < 0.05 versus control.

In a CyQUANT proliferation assay, 20 h exposure to the drugs induced only a modest, non-significant increase in HPMEC-ST1.6R cell number. In contrast, in a scratch (wound-healing assay), budesonide significantly accelerated closure of the endothelial monolayer over 4 h (≈14% versus ≈10% reduction in wound area in control; *p* < 0.05, *n* = 3; Figure 4). Enhanced monolayer repair in the absence of increased proliferation indicates that budesonide accelerates endothelial wound closure by promoting reparative cell behavior rather than stimulating cell proliferation.

### 2.5. Budesonide and Formoterol Alter Endothelial Network Organisation on Matrigel

**Figure 5:**
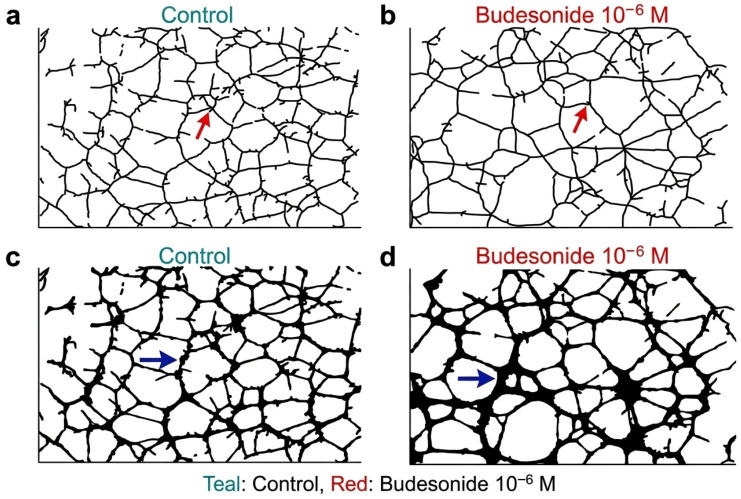
Representative endothelial tube networks on Matrigel. Traced outlines of HPMEC-ST1.6R networksunder control (**a**,**c**) and budesonide (**b**,**d**) conditions. Red arrows indicate reduced junctions and blue arrows indicate increased tubule thickness under budesonide treatment.

**Figure 6:**
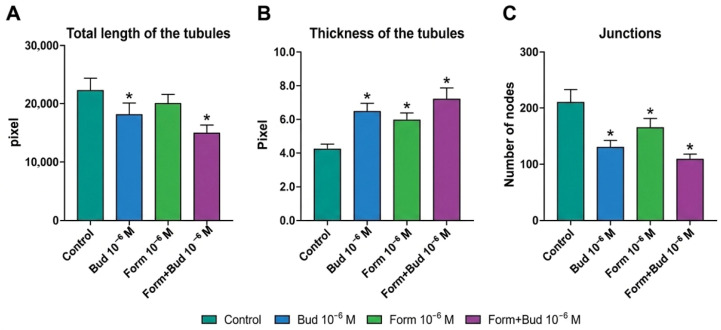
Quantification of Matrigel tube formation. (**A**) Total tubule length, (**B**) mean tubule thickness, and (**C**) number of junctions/nodes (pixels) for control, Bud, Form and Bud + Form (each 10^−6^ M). Total length and junctions were significantly reduced and thickness significantly increased by all treatments, without additional effects. Mean ± SEM, *n* = 3. * *p* < 0.05 versus control.

Endothelial network formation was modelled by seeding HPMEC-ST1.6R cells on Matrigel, where they rapidly assemble into capillary-like tubular networks. As shown in representative skeletonized network tracings, budesonide, formoterol, and their combination (10^−6^ M) altered network architecture compared with controls (Figure 5). This latter included reduced branching complexity, shorter interconnecting segments, and increased tube size. Quantitative analysis was performed using an automated image-processing pipeline based on CellProfiler [21], enabling objective extraction of vascular network parameters from fluorescence microscopy images. This approach provides unbiased measurements of key angiogenic features, including total tubule length, number of junctions/nodes, and mean tubule thickness, derived from skeletonized network maps. Consistent with these analyses, all three treatments significantly reduced total tubule length and the number of junctions/nodes (control ≈210), while significantly increasing mean tubule thickness (*p* < 0.05, *n* = 3; Figure 6). The combination of budesonide and formoterol did not exceed the effects of either agent alone.

### 2.6. Budesonide Preserves Endothelial Spheroid Integrity and Reduces Stress-Induced Cell Death

To investigate endothelial differentiation and integrity in a three-dimensional context, HPMEC-ST1.6R cells were cultured as spheroids by aggregation in a Methocel-containing suspension, forming compact spheroids with a continuous outer endothelial cell layer [22].

**Figure 7:**
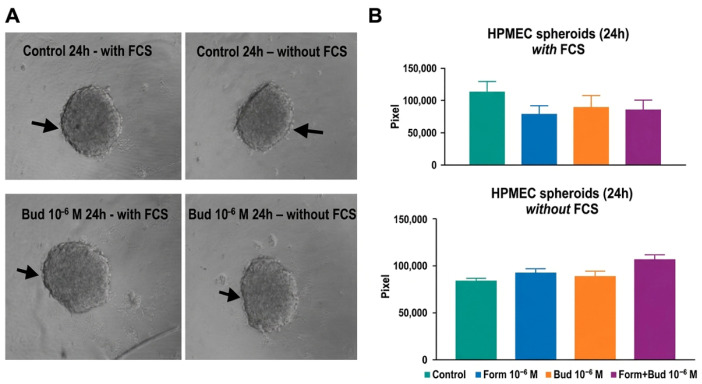
Endothelial spheroid size at 24 h is unaffected by treatment. Cross-sectional spheroid area (pixels) of HPMEC-ST1.6R spheroids after 24 h, in (**A**) serum-containing (with FBS) and (**B**) serum-free (without FBS) medium, under Bud, Form and Bud + Form (each 10^−6^ M). No significant differences; endothelial monolayer differentiation was preserved (black arrows indicate spheroids). Mean ± SEM, *n* = 3.

**Figure 8:**
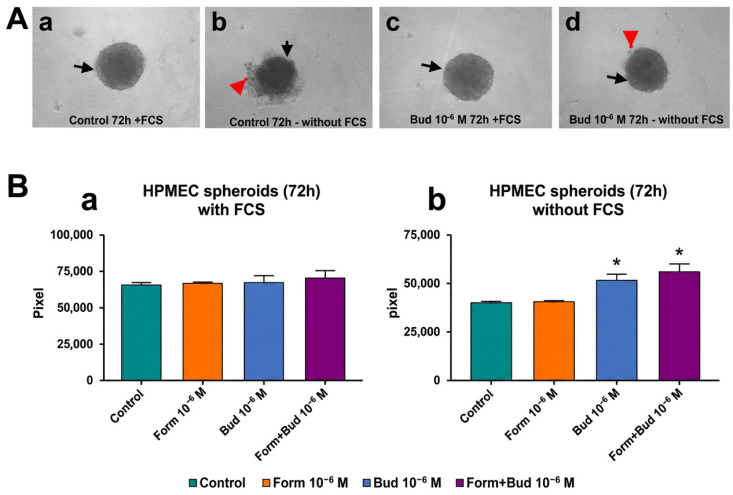
Budesonide reduces spheroid cell death at 72 h. HPMEC-ST1.6R endothelial spheroids were cultured for 72 h and analysed for size and dead-cell accumulation. (**A**) Representative phase-contrast micrographs of spheroids: (**a**) control, 72 h, serum-containing medium (+FCS); (**b**) control, 72 h, serum-free medium (without FCS); (**c**) budesonide (10–6 M), 72 h, serum-containing medium (+FCS); (**d**) budesonide (10–6 M), 72 h, serum-free medium (without FCS). Black arrows indicate intact spheroids; red arrowheads indicate dead cells accumulating at the spheroid periphery under serum-free conditions. (**B**) Quantification of cross-sectional spheroid area (pixels) for control, Form (10–6 M), Bud (10–6 M) and Bud + Form (10–6M): (**a**) in serum-containing medium (with FCS), showing no significant differences between groups; (**b**) in serum-free medium (without FCS), where budesonide and Bud + Form significantly reduced dead-cell accumulation and preserved larger spheroids relative to control. The protective effect of the drugs is not seen in serum-containing medium (**A**). Mean ± SEM, *n* = 3. * *p* < 0.05 versus control.

Spheroid formation and initial spheroid size at 24 h were not significantly affected by budesonide or formoterol, either in the presence or absence of fetal calf serum (FCS) (Figure 7). Over 72 h, spheroids progressively decreased in size and showed increased accumulation of dead cells at the periphery, particularly under serum-free conditions. Treatment with budesonide, alone or in combination with formoterol, significantly reduced peripheral cell death and preserved spheroid size compared with control conditions (*p* < 0.05; Figure 8). Importantly, this protection was consistent with reduced caspase-3/7 activation and improved viability observed in monolayer cultures (Figure 3), suggesting that budesonide attenuates stress-induced apoptotic loss in pulmonary microvascular endothelial cells under these experimental conditions.

### 2.7. Fluticasone/Salmeterol Reproduces ICS-Associated Suppression of the Epithelial Secretome

**Figure 9:**
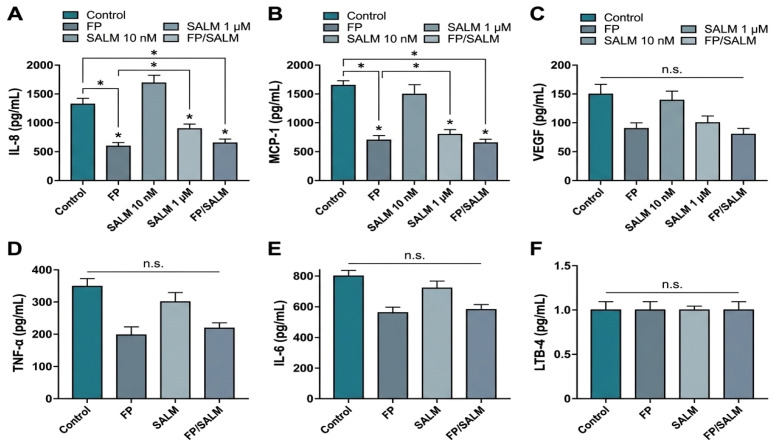
Fluticasone/salmeterol reproduces ICS-associated suppression of the epithelial inflammatory and angiogenesis-associated secretome. Secreted cytokine concentrations (pg/mL, ELISA) from unstimulated A549 alveolar epithelial cells (panels (**A**) IL-8, (**B**) MCP-1, (**C**) VEGF) and J774A.1 macrophages (panels (**D**) TNF-α, (**E**) IL-6, (**F**) LTB-4) after 24 h treatment with fluticasone propionate (FP), salmeterol (SALM) or the FP/SALM combination, each applied at 10 nM and 1 µM. IL-8 and MCP-1 were significantly reduced by FP and FP/SALM; VEGF, TNF-α and IL-6 were reduced without reaching significance; LTB-4 was unchanged. The response was corticosteroid-dominated, with salmeterol alone largely inactive and no additional activity of the combination. Mean ± SEM, *n* = 3. * *p* < 0.05 versus control.

To determine whether suppression of the epithelial secretome reflects a general corticosteroid-associated effect rather than a budesonide-specific property, we analyzed an independent dataset using a second ICS/LABA combination, fluticasone propionate (FP) and salmeterol (salmeterol, SALM), in A549 alveolar epithelial cells and J774A.1 macrophages. Cells were treated for 24 h with FP (10 nM) alone or in combination with SALM (1 µM). In unstimulated A549 cells, FP and FP/SALM significantly reduced secretion of IL-8 (≈1330 to ≈500–700 pg/mL) and MCP-1 (≈1650 to ≈600–800 pg/mL; *p* < 0.05, *n* = 3), thereby reproducing the suppression observed with budesonide. VEGF secretion was also reduced by FP and FP/SALM (≈150 to ≈70–100 pg/mL), although this effect did not reach statistical significance. In J774A.1 macrophages, FP and FP/SALM reduced TNF-α (≈350 to ≈180–240 pg/mL) and IL-6 (≈800 to ≈550–600 pg/mL), without statistical significance, whereas LTB4 levels remained unchanged (Figure 9). Consistent with the budesonide/formoterol data, the response was primarily driven by the corticosteroid component: salmeterol alone was largely inactive and at 10 nM transiently increased IL-8 and MCP-1, the combination did not exceed FP alone, and no additional biological effects were observed.

**Figure 10:**
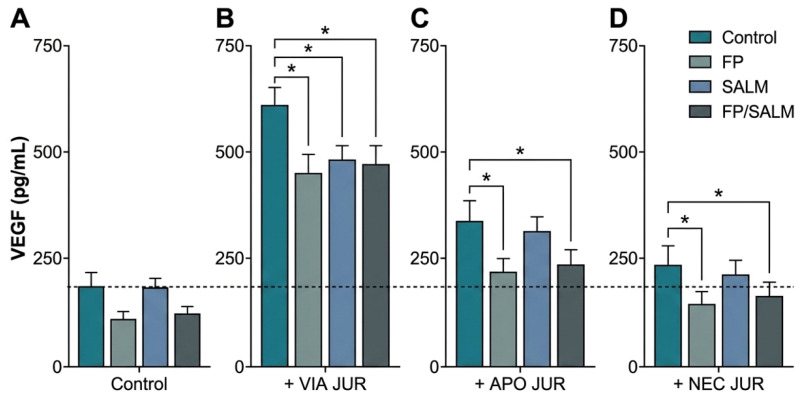
Inhaled corticosteroids modulate epithelial VEGF secretion in a cell-context-dependent manner. VEGF concentrations (pg/mL, ELISA) in A549 alveolar epithelial cells treated for 24 h with fluticasone propionate (FP), salmeterol (SALM) or the FP/SALM combination, and co-incubated with Jurkat T cells under four conditions: (**A**) basal control without added Jurkat cells; (**B**) co-incubation with viable Jurkat cells (+VIA JUR); (**C**) co-incubation with apoptotic Jurkat cells (+APO JUR); and (**D**) co-incubation with necrotic Jurkat cells (+NEC JUR). Within each panel, bars represent the untreated control and the FP, SALM and FP/SALM treatment groups. The dashed horizontal line marks the basal VEGF level of unstimulated A549 cells. Co-incubation with viable Jurkat cells sharply increased VEGF secretion, which was significantly reduced by FP and the FP/SALM combination, whereas the apoptotic and necrotic conditions produced lower VEGF that was further attenuated by corticosteroid treatment. Mean ± SEM, *n* = 3. * *p* < 0.05 versus the corresponding co-incubation control.

**Figure 11:**
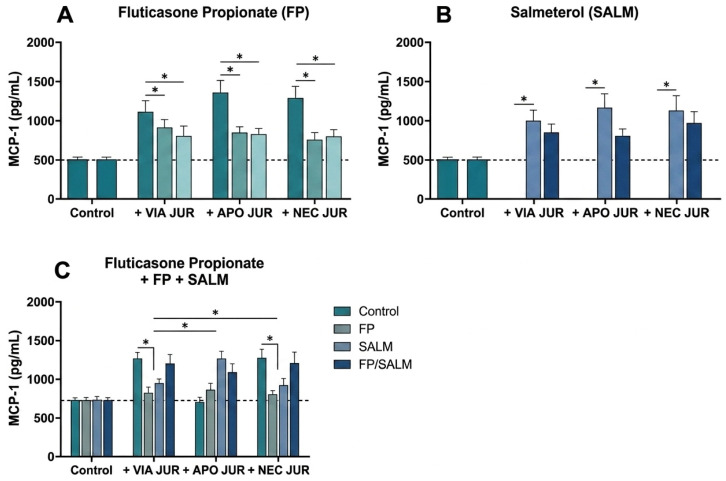
Fluticasone propionate suppresses macrophage MCP-1 secretion across cellular contexts in a concentration-dependent manner. MCP-1 concentrations (pg/mL, sandwich ELISA) in J774A.1 macrophages after a single 24 h treatment (a dose–response experiment performed at one time point, not a time–course study). Cells were treated with fluticasone propionate (FP; panel (**A**)), salmeterol (SALM; panel (**B**)) or the FP/SALM combination (panel (**C**)), each applied at 10 nM and 1 µM, and assessed under four cellular contexts: basal A549/macrophage co-culture without added lymphocytes (Control) and co-incubation with viable (+VIA JUR), apoptotic (+APO JUR) or necrotic (+NEC JUR) Jurkat T cells. In panel (**A**), the bars within each context represent increasing FP concentrations (untreated control, 10 nM and 1 µM FP); panels (**B**,**C**) follow the same four-context layout, with bars coloured by treatment group (Control, FP, SALM, FP/SALM). The dashed horizontal line in each panel marks the basal MCP-1 level of unstimulated J774A.1 macrophages. FP lowered MCP-1 secretion in a concentration-dependent manner across all contexts, salmeterol alone was largely inactive, and the FP/SALM combination did not exceed FP alone, consistent with a corticosteroid-dominated response and no additional activity of the LABA. Mean ± SEM, *n* = 3. * *p* < 0.05 versus the corresponding within-context control.

Because epithelial–endothelial cross-talk in the injured lung is influenced by the clearance of apoptotic and necrotic cells, we next examined cytokine secretion in A549 and J774A.1 cells co-incubated with viable, apoptotic, or necrotic Jurkat T cells. Co-incubation with viable Jurkat cells markedly increased A549 VEGF secretion (≈180 to ≈600 pg/mL; *p* < 0.05) while reducing IL-8 and MCP-1, indicating that the cellular context critically determines the direction of VEGF regulation. Across these conditions, FP and SALM consistently acted in the same direction, with no consistent evidence for additional activity of the LABA component, apart from TNF-α release from J774A.1 cells co-incubated with necrotic Jurkat cells (Figure 10 and Figure 11).

Taken together, the budesonide/formoterol results indicate that the suppression of the IL-8/MCP-1/VEGF axis is a shared, corticosteroid-associated response across two structurally distinct ICS/LABA combinations in alveolar epithelial cells, independent of apoptotic or necrotic co-stimulation.

## 3. Discussion

The principal finding of this study is that budesonide exerts distinct effects on the pulmonary microvascular endothelium by modulating endothelial network formation while preserving endothelial viability under stress. In the Matrigel assay, budesonide (and formoterol) reduced total tubule length and the number of junctions, while increasing tubule thickness. In alveolar epithelial cells, budesonide also reduced the secretion of angiogenesis-associated mediators VEGF, IL-8 and MCP-1. These findings are consistent with clinical and biopsy studies showing that inhaled corticosteroids reduce pathological airway angiogenesis and VEGF expression in airway remodeling in asthma [17,18,19]. Together, these results suggest that the endothelial network formation and endothelial survival are distinct biological processes that are differentially regulated by budesonide.

We also show that suppression of the epithelial secretome is not limited to a single drug combination. An independent dataset obtained using a second, structurally distinct ICS/LABA combination, fluticasone propionate and salmeterol, reproduced the key epithelial phenotype, showing significant reductions in IL-8 and MCP-1, with a downward trend in VEGF, TNF-α, and IL-6 in both A549 and J774A.1 cells. Two unrelated corticosteroids (budesonide and fluticasone propionate), each combined with a different β2-agonist (formoterol and salmeterol), produced a consistent effect on the IL-8/MCP-1/VEGF axis, supporting a shared corticosteroid-associated response rather than an idiosyncratic property of a single compound. We deliberately avoid referring to this as a class effect, as two compounds are insufficient to support such a conclusion. Moreover, endothelial endpoints (apoptosis, Matrigel, and spheroid assays) were assessed only for budesonide, whereas the second dataset was restricted to epithelial secretory responses. Nevertheless, the corticosteroid-dominant pattern, with no additional contribution from the LABA, was consistent across both experimental approaches, supporting a notion that these effects are primarily glucocorticoid-driven.

The observation that viable, but not apoptotic, lymphocytes markedly increased epithelial VEGF secretion further suggests that the cellular microenvironment, including the dying cells and their impaired clearance in COPD, may contribute to the regulation of angiogenic signaling [10,11,12]. Elevated airway VEGF is a consistent feature of obstructive airway disease [19,23]. Accordingly, the reduction in VEGF, together with IL-8 and MCP-1, induced by budesonide may limit pathological pro-angiogenic signaling while preserving endothelial viability, as demonstrated in our cytoprotecting assays.

At first glance, the reduction in VEGF together with decreased endothelial network formation appears inconsistent with the vascular hypothesis of emphysema, which proposes that loss of VEGF signaling promotes endothelial apoptosis and alveolar destruction [3,4,7]. However, our findings suggest that these processes can be uncoupled. Although budesonide reduced paracrine VEGF production and decreased network complexity, it also protected endothelial cells from serum deprivation/UV-induced apoptosis, reduced caspase-3/7 activity, accelerated wound closure, and preserved the integrity of three-dimensional endothelial spheroids. These results indicate that reduced angiogenesis does not necessarily lead to endothelial injury or cell death. Instead, the observed changes in network morphology may reflect altered endothelial organization rather than toxicity. Specifically, the combination of fewer branch points and increased tubule thickness is consistent with a transition from highly sprouting, immature networks to fewer, larger, and more stable vascular structures. While this interpretation suggests that budesonide promotes vascular stabilization rather than endothelial damage, the underlying mechanisms remain uncertain because markers of endothelial maturation and angiogenic signaling were not directly examined.

The endothelial-protective phenotype may be relevant to COPD, although this link remains hypothetical rather than mechanistically demonstrated. Because endothelial apoptosis in the alveolar septum can initiate emphysema [3,4,8], an agent that reduces caspase activation and preserves endothelial survival under stress could, in principle, counter cellular events that contribute to alveolar loss. It is important to emphasize that these experiments used immortalised cell lines under artificial stressors (serum deprivation, UV exposure) and did not include COPD-derived cells, cigarette-smoke exposure, or in vivo models; the COPD relevance therefore represents an extrapolation rather than a proven disease mechanism. Our three-dimensional spheroid data are nonetheless informative: under serum withdrawal, an established trigger of endothelial apoptosis [5,6], budesonide reduced peripheral cell death and preserved spheroid integrity, recapitulating the protective effects observed in monolayer cultures.

A consistent theme across all endpoints was the dominant effect of budesonide and the absence of a clear independent effect of formoterol. No additional biological activity attributable to the LABA component was observed across the endothelial endpoints, and the combination largely mirrored corticosteroid alone. In this system, formoterol acted as a negative comparator, indicating that the observed effects are primarily driven by the corticosteroid. Previous reports of additive or synergistic ICS/LABA interactions have largely focused on airway smooth muscle, bronchial epithelium, or inflammatory signaling, where β2-agonists can enhance glucocorticoid receptor nuclear translocation and amplify anti-inflammatory gene regulation [13,16]. In contrast, our data indicate that endothelial responses are largely driven by the corticosteroid component, with minimal contribution from the β2-agonist under the conditions used. This suggests that ICS/LABA interactions are cell type- and endpoint-dependent rather than universally applicable. A plausible cellular basis is that the pulmonary microvascular endothelium differs from airway smooth muscle, epithelium, and fibroblasts in β2-adrenergic receptor expression and coupling to downstream cAMP/PKA signalling, limiting a functional substrate for independent β2-agonist activity in this cell type. Accordingly, endothelial processes such as network formation and intrinsic apoptosis appear to be predominantly glucocorticoid-driven. This suggests that the established clinical benefit of adding a LABA in COPD likely reflects effects in other lung compartments rather than direct modulation of the pulmonary microvascular endothelium [14,15]. The restoration of apoptotic-cell clearance observed after budesonide pre-incubation, but not co-incubation, further indicates that the timing of glucocorticoid exposure regulates efferocytosis, which is relevant to impaired clearance in COPD [10,11,12].

A clinically important counterpoint is the well-recognised association between long-term inhaled corticosteroid use and pneumonia in COPD. Real-world studies indicate that prolonged ICS exposure approximately doubles the risk of pneumonia and other adverse events [24]. Narrative reviews identify fluticasone-containing regimens as carrying the highest pneumonia risk signal [25]. Our in vitro findings that two ICS/LABA combinations suppress a pro-angiogenic epithelial secretome while protecting the pulmonary microvascular endothelium describe a cell-level effect that coexists with the recognised clinical liability of ICS. These data do not support broader ICS use; rather, at the cellular level they may help to characterise a possible endothelial component of the ICS risk–benefit balance in emphysema, a link that remains hypothetical and is not established by the present in vitro data. Notably, the endothelial-protective phenotype observed here contrasts with the pro-apoptotic, anti-angiogenic effects of glucocorticoids reported in other vascular beds. In bone microvascular endothelial cells, glucocorticoids inhibit PI3K/Akt signalling, promote apoptosis, and impair tube formation and migration, with disruption of this pathway contributing to steroid-induced osteonecrosis [26,27]. That budesonide preserved viability, reduced caspase-3/7 activity, and accelerated repair in pulmonary microvascular endothelium may indicate a tissue-specific divergence in glucocorticoid–endothelial coupling. Importantly, we did not assess PI3K/Akt activity, Bcl-2 family members, or related signaling intermediates; these pathways are referenced only in the context of existing literature and were not experimentally tested. Whether pulmonary endothelial responses reflect differential regulation of survival pathways compared with other vascular beds remains an open mechanistic question not resolved by the present functional data.

Several limitations should be acknowledged, given the exploratory and in vitro nature of this study. First, the work relies on transformed or immortalized cell lines (A549 and HPMEC-ST1.6R), which do not fully recapitulate primary epithelial or microvascular endothelial biology, and findings will require validation in primary cells and in vivo models. Second, most experiments were performed with *n* = 3 biological replicates, limiting statistical power and the interpretation of non-significant trends. Third, the fluticasone/salmeterol dataset strengthens generalisability but was restricted to epithelial and macrophage secretory responses, without endothelial functional assays; endothelial conclusions are therefore based on budesonide data alone. Fourth, drug exposures were not systematically dose–response tested, and the physiological relevance of the concentrations used remains uncertain. Fifth, apoptotic stimuli were non-specific (serum deprivation and UV irradiation) and do not fully model COPD-related injury. Finally, mechanistic pathways downstream of glucocorticoid receptor activation were not assessed, so mechanistic interpretations remain speculative. Collectively, these limitations indicate that the findings should be interpreted as hypothesis-generating and require confirmation in more physiologically relevant models.

## 4. Materials and Methods

### 4.1. Cell Culture

The immortalized human pulmonary microvascular endothelial cell line HPMEC-ST1.6R was kindly provided by Prof. C. J. Kirkpatrick (Institute of Pathology, Johannes Gutenberg University, Mainz, Germany) and was generated and characterized as described [20]. Cells were cultured in Medium 199 supplemented with 20% fetal bovine serum (FBS), 50 µg/mL endothelial cell growth supplement (ECGS), 25 µg/mL heparin, 2 mM GlutaMAX and 1% penicillin/streptomycin, and retained endothelial identity (PECAM-1/CD31, von Willebrand factor, ICAM-1, VCAM-1, E-selectin). The human alveolar epithelial cell line A549 (DSMZ ACC 107; DSMZ, Braunschweig, Germany) was maintained in DMEM/RPMI with 10% FBS. Jurkat T cells were used as apoptotic targets. THP-1 monocytes were differentiated to macrophages with phorbol 12-myristate 13-acetate (PMA, 100 nM) for 72 h; the murine macrophage line J774A.1 served as professional phagocytes. All cells were maintained at 37 °C in 5% CO_2_.

### 4.2. Test Substances

Budesonide and formoterol (budesonide, Sigma-Aldrich, St. Louis, MO, USA cat. no. B7777; formoterol fumarate, cat. no. F9552) were dissolved as dimethyl sulfoxide (DMSO) stock solutions (final DMSO concentration < 0.1%) and applied at a final concentration of 10^−6^ M, alone or in combination, for 24 h unless otherwise stated. In the independent convergence dataset, fluticasone propionate (FP) and salmeterol (SALM) (fluticasone propionate, Sigma-Aldrich (St. Louis, MO, USA) cat. no. F9428; salmeterol xinafoate, cat. no. PHR1947) were applied at 10 nM and 1 µM, alone or in combination (FP/SALM), for 24 h. Vehicle-treated cells served as controls throughout.

### 4.3. Induction and Detection of Apoptosis

Apoptosis in Jurkat target cells was induced by UV irradiation (100 mJ/cm^2^). Necrotic Jurkat cells were generated by heating the cell suspension at 56 °C for 30 min, a standard method for primary necrosis. For endothelial apoptosis, HPMEC-ST1.6R cells were cultured in suspension dishes under FBS deprivation for 6 h, with or without additional UV irradiation. Apoptotic and dead cells were identified by fluorescence microscopy using Hoechst-33342 with Sytox Green. Caspase-3/7 activity was quantified with the Apo-ONE Homogeneous Caspase-3/7 assay (Z-DEVD-rhodamine 110 substrate; Promega, Madison, WI, USA) at 6 h and 20 h.

### 4.4. Phagocytosis (Efferocytosis) Assay

Phagocytes (J774A.1, THP-1-derived macrophages, A549, HPMEC-ST1.6R) were pre-incubated with budesonide, formoterol or their combination for 24 h. Fluorescently labeled apoptotic Jurkat T cells were then added at an effector:target ratio of 5:1 (apoptotic Jurkat cells:phagocytes), and after co-incubation for 24 h and washing of unbound targets thepercentage of phagocytosis-positive cells was determined by fluorescence microscopy.

### 4.5. Cytokine ELISA

For the budesonide/formoterol experiments, concentrations of IL-8, MCP-1 and VEGF in A549 culture supernatants were measured by sandwich ELISA after 24 h treatment according to the manufacturer’s instructions. For the convergence (fluticasone/salmeterol) experiments, A549 alveolar epithelial cells and J774A.1 macrophages were treated with FP, SALM or FP/SALM (10 nM and 1 µM, 24 h), either unstimulated or co-incubated with viable, apoptotic or necrotic Jurkat T cells. Viable Jurkat cells were untreated cells harvested from log-phase culture. Supernatant IL-8, MCP-1, VEGF, TNF-α, IL-6 and LTB-4 were quantified by sandwich ELISA using DuoSet ELISA kits (R&D Systems, Minneapolis, MN, USA) according to the manufacturer’s instructions. All measurements were performed in at least three independent experiments.

### 4.6. Proliferation and Wound-Healing Assays

Proliferation of HPMEC-ST1.6R cells was assessed with the CyQUANT assay after 20 h. Migration was assessed in a scratch wound-healing assay; the cell-free area was imaged at 0 h and 4 h and the reduction in wound area relative to the initial (0 h) wound area was quantified with CellProfiler (version 4.2.6) [21].

### 4.7. Matrigel Tube-Formation Assay

HPMEC-ST1.6R cells were seeded on growth-factor-containing Matrigel and allowed to form capillary-like networks, which developed within 4–6 h. After treatment, networks were imaged and quantified for total tubule length, mean tubule thickness, and number of junctions/nodes by automated image analysis with CellProfiler [21].

### 4.8. Three-Dimensional Spheroid Assay

Endothelial spheroids were generated by the hanging-drop/Methocel method of Korff and Augustin [22] (≈750 cells per spheroid). Spheroids were cultured for 24 h (size analysis) or 72 h with or without FBS (viability analysis), stained with Sytox Green to identify dead cells, imaged, and quantified for cross-sectional area and dead-cell number.

### 4.9. Image Analysis and Statistics

Quantitative image analysis was performed with CellProfiler [21]. Data are expressed as mean ± SEM from at least three independent biological experiments (*n* denotes independent experiments, not technical replicates, images, or individual spheroids). Distribution was assessed with the Kolmogorov–Smirnov test; group comparisons used Student’s *t*-test or the Mann–Whitney U test for two groups and one-way ANOVA with the Student–Newman–Keuls post-test for multiple groups (GraphPad Prism version 10.2.3 (GraphPad Software, Boston, MA, USA)). We note that Dunnett’s or Tukey’s corrections provide more conservative control of the family-wise error rate for multi-group comparisons, and that the factorial designs underlying Figure 10 and Figure 11 would be well suited to two- or three-way ANOVA in future analyses. A value of *p* < 0.05 was considered statistically significant.

## 5. Conclusions

In our in vitro models, budesonide exerts coordinated but functionally distinct effects on pulmonary microvascular endothelium, altering network organisation while preserving endothelial viability, accelerating repair, maintaining spheroid integrity, and enhancing apoptotic cell clearance after pre-incubation. A second, structurally unrelated ICS/LABA combination (fluticasone propionate/salmeterol) independently reproduced suppression of the epithelial secretome, supporting a corticosteroid-associated response rather than a compound-specific effect. In both datasets, the β2-agonist contributed little additional biological activity. Overall, these findings suggest that endothelial survival and network organization represent partially dissociable glucocorticoid-regulated processes in vitro and may provide a preliminary cellular framework for exploring the endothelial effects of ICS relevant to emphysema. However, confirmation in primary cells and in vivo models, alongside dose–response and mechanistic studies, will be required for clinical translation.

## Data Availability

The original contributions presented in this study are included in the article. Further inquiries can be directed to the corresponding author(s).
